# Metabolic Alterations in Inherited Cardiomyopathies

**DOI:** 10.3390/jcm8122195

**Published:** 2019-12-12

**Authors:** Claudia Sacchetto, Vasco Sequeira, Edoardo Bertero, Jan Dudek, Christoph Maack, Martina Calore

**Affiliations:** 1 IMAiA—Institute for Molecular Biology and RNA Technology, Faculty of Health, Universiteitssingel 50, 6229ER Maastricht, The Netherlands; c.sacchetto@maastrichtuniversity.nl; 2 Medicine and Life Sciences, Faculty of Science and Engineering, Universiteitssingel 50, 6229ER Maastricht, The Netherlands; 3 Department of Biology, University of Padova, via Ugo Bassi 58B, 35121 Padova, Italy; 4 Department of Translational Science, Comprehensive Heart Failure Center, University Clinic Würzburg, Am Schwarzenberg 15, 9708 Würzburg, Germany; Sequeira_V@ukw.de (V.S.); Bertero_E@ukw.de (E.B.); Dudek_J@ukw.de (J.D.)

**Keywords:** inherited cardiomyopathies, mitochondria, cardiac metabolism

## Abstract

The normal function of the heart relies on a series of complex metabolic processes orchestrating the proper generation and use of energy. In this context, mitochondria serve a crucial role as a platform for energy transduction by supplying ATP to the varying demand of cardiomyocytes, involving an intricate network of pathways regulating the metabolic flux of substrates. The failure of these processes results in structural and functional deficiencies of the cardiac muscle, including inherited cardiomyopathies. These genetic diseases are characterized by cardiac structural and functional anomalies in the absence of abnormal conditions that can explain the observed myocardial abnormality, and are frequently associated with heart failure. Since their original description, major advances have been achieved in the genetic and phenotype knowledge, highlighting the involvement of metabolic abnormalities in their pathogenesis. This review provides a brief overview of the role of mitochondria in the energy metabolism in the heart and focuses on metabolic abnormalities, mitochondrial dysfunction, and storage diseases associated with inherited cardiomyopathies.

## 1. Introduction 

**Figure 1:**
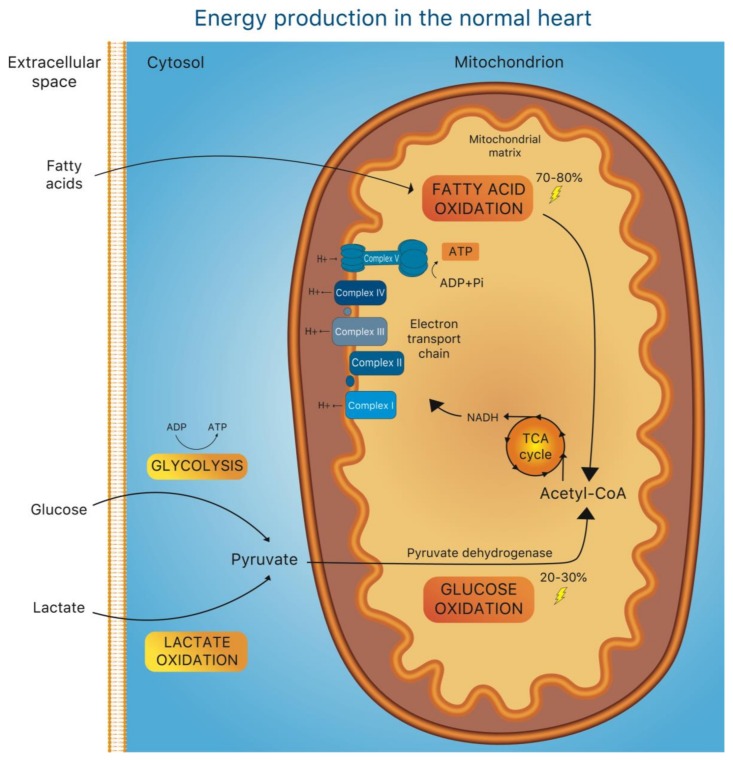
Energy production in the normal heart. Overview of the central metabolic pathways contributing to ATP production in the heart. Mitochondrial fatty acid oxidation (FAO) is the main source of energy (70%–80%). The remaining 20%–30% of ATP production largely derives from glucose oxidation. During this process, the pyruvate produced in the cytosol as result of glycolysis and lactate oxidation is transferred to the mitochondria and converted to acetyl-CoA by the pyruvate dehydrogenase complex (PDC). Acetyl-CoA, which is also the final product of fatty acid oxidation, enters the tricarboxylic acid cycle (TCA cycle) promoting the production of nicotinamide adenine dinucleotide (NADH), and thus providing a source of electrons for the electron transport chain (ETC), located at the inner mitochondrial membrane. Within the ETC, each complex contributes to the creation of a proton gradient fundamental to provide sufficient energy to generate ATP from adenosine diphosphate (ADP).

The heart has a very high energy demand imposed by the maintenance of specialized cellular processes, such as intracellular ion homeostasis and contractile function of cardiomyocytes. Due to the limited capacity for substrate storage in the heart, the balance between substrate demand and oxidation is pivotal to normal cardiac function and is finely regulated at multiple levels [1]. Under normal conditions, adenosine triphosphate (ATP) is produced predominantly (70%–80%) from fatty acid (FA) oxidation in mitochondria, while the remaining 20% to 30% derive from oxidation of glucose and lactate, with a minor contribution of ketone body and amino acid oxidation [1,2], (Figure 1). Of note, glucose oxidation is energetically more efficient than FA β-oxidation (FAO), since more ATP is produced for any given amount of molecular oxygen (O2) consumed (3.17 ATP/O2 formed from glucose vs. –2.5 ATP/O2 formed from FA) [3]. A rather simplistic view proposes that the heart’s metabolic over-reliance on FA depends on a much larger availability of body fat reserves―at any given moment―than those available from glucose [4].

Acute increases in cardiac workload are matched by a rapid increase in substrate supply and ATP regeneration via: (1) increases in coronary flow and oxygen utilization, but also (2) the metabolic shift from FA towards glucose oxidation (“the Randle cycle”). 

It is generally agreed on that heart failure (HF) is associated with a shift from the preferential utilization of FA towards glucose oxidation for ATP production [5]. However, it is still unresolved whether this shift is adaptive or maladaptive to the heart, but this may largely depend on several factors, including the degrees of workload and wall stress, as well as the stage of the disease. More importantly, if glucose oxidation indeed represents a metabolic “reserve” mechanism to cope with acute―but short-term―increases in cardiac workload and stress, its prolonged utilization likely indicates a long-term maladaptive response.

**Figure 2:**
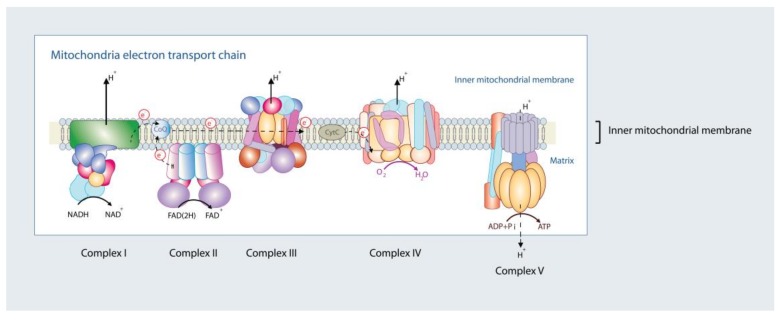
Mitochondrial electron transport chain. Complex I (NADH dehydrogenase), Complex III (cytochrome b–c1 complex), Complex IV (cytochrome c oxidase), and Complex V (ATP synthase) span the inner mitochondrial membrane. Complex II is non-membrane spanning. Reduced forms of NADH (complex I) and FAD(2H) (complex II) donate electrons (e^-^) to the transport chain via complex I and/or complex II, respectively, which are sequentially transferred to electron carriers, including the lipid soluble coenzyme Q (CoQ), complex III, cytochrome *c* (CytC), and complex IV. Complex IV accepts e^-^ from the electron transport chain and reduces molecular oxygen (O_2_) into water (H_2_O). As e^-^ pass the electron transfer chain, protons (H^+^) are pumped across the mitochondrial matrix to the inner mitochondrial space (at complexes I, III, and IV; complex II lacks a proton pumping mechanism), responsible for establishing an electrochemical proton gradient at the inner mitochondrial membrane. The creation of the electrochemical proton gradient forces protons back inside the matrix at complex V, which uses the H^+^ gradient energy to regenerate ATP from ADP (and Pi). The electron transport chain couples the rate of ATP regeneration by the electrochemical proton gradient-coupled oxidative phosphorylation. Under physiological conditions, approximately up to 5% of O_2_ in cells is converted to reactive oxygen species (ROS), with complexes I and III the main sites for ROS production.

On the one hand, derangements in mitochondrial oxidative metabolism can cause energetic deficit and oxidative stress in cardiomyocytes, ultimately fueling a vicious cycle that promotes the deterioration of cardiac function. On the other hand, ATP deficiency can also represent a consequence of HF, since it can result from the maladaptive remodeling of the cellular processes regulating energy production in mitochondria, such as intracellular calcium (Ca2+) homeostasis and the expression of metabolic genes [5,6,7,8]. Ca2+ shuttling between mitochondria and the cytosol is pivotal to adapt mitochondrial ATP production to the constantly changing energy requirements imposed by the processes of excitation-contraction coupling [7,9]. During an increase in cardiac workload, sympathetic activation increases heart rate and cellular contractility. The increase in cardiac contractile performance is mainly achieved via the activation of the cyclic adenosine monophosphate (cAMP)-protein kinase A (PKA) axis, which (i) enhances Ca2+ entry via the sarcolemmal L-type Ca2+ channels, triggering even larger releases of Ca2+ from the sarcoplasmic reticulum (SR), (ii) desensitizes the contractile apparatus to Ca2+, and (iii) accelerates Ca2+ reuptake into the SR during diastole [10,11]. The concomitant increase in heart rate and contractility increases ATP consumption, which needs to be matched by an increase in ADP phosphorylation in mitochondria. This metabolic adaptation is driven by the parallel activation of mitochondrial oxidative metabolism by ADP, which is delivered to the respiratory chain via the creatine and adenosine nucleotide shuttles, and Ca2+, which accumulates inside mitochondria as a result of the increase in amplitude and frequency of cytosolic Ca2+ transients during sympathetic activation [7,12]. In the mitochondrial matrix, Ca2+ stimulates the rate-limiting reactions of the Krebs cycle and thereby, accelerates the regeneration of the reduced form of nicotinamide adenine dinucleotide (NADH) [7,9], which fuels oxidative phosphorylation by donating electrons to the respiratory chain complexes embedded in the inner mitochondrial membrane (Figure 2) [7,13]. Increases in mitochondrial matrix Ca2+ that exceed the physiological levels, for instance during myocardial ischemia and reperfusion, should be avoided since this activates the mitochondrial permeability transition pore, a large conductance channel that causes cellular death by apoptosis and/or necrosis [14].

Perturbations of these processes occur together with profound structural cardiac remodeling (e.g., hypertrophy, dilation) and concomitant deterioration of cardiac performance, two of the hallmarks of HF. HF is one of the major causes of morbidity and mortality worldwide [15] and can be determined by both inherited and non-inherited causes. Although the origin of inherited disorders can be traced to a single genetic mutation, the development and progression of a cardiomyopathic phenotype depends on the complex interaction between cellular signaling pathways, environmental stressors, and individual genotype. In the last years, due to advances in imaging, genetics and genomics, important steps in understanding the major forms of cardiomyopathy have been made [16]. Besides the disease-specific perturbations, inherited cardiomyopathies show also alterations in myocardial energy metabolism that concur to the progression of HF with or without the involvement of other organs [7,12]. In this review, we focus on the derangements in cardiac metabolism in inherited cardiomyopathies (Table 1).

## 2. Altered Mechano-Chemo-Transduction and Oxidative Stress in Heart Failure 

Heart failure per se can occur as heart failure with reduced (HFrEF) or preserved ejection fraction (HFpEF). While the mechanisms of HFpEF are still incompletely resolved, involving alterations of myofilaments and the extracellular matrix that compromise diastolic function [17], the development of HFrEF is caused by derangements of excitation-contraction coupling, where a decreased Ca2+ load of the sarcoplasmic reticulum (SR), with subsequently reduced amplitudes of cytosolic Ca2+ transients is a major cause of systolic dysfunction [18,19]. These alterations in intracellular ion handling disrupt the process of energy supply-and-demand matching by impairing the accumulation of Ca2+ inside mitochondria. Besides the reduced SR Ca2+ release, which hampers mitochondrial Ca2+ uptake [20], HF is also characterized by an increase in the intracellular sodium (Na+) concentration of cardiac myocytes [9,13]. Because Ca2+ efflux from mitochondria is mediated by a Na+/Ca2+ exchanger (NCLX), elevations of cytosolic Na+ accelerate Ca2+ extrusion from the mitochondrial matrix, consequently hindering the Ca2+-dependent stimulation of the Krebs cycle [9].

Oxidative stress is a hallmark of HF, and mitochondria are the main source of reactive oxygen species (ROS) in cardiac myocytes [21,22]. In the failing heart, the emission of ROS from mitochondria is the result of their increased production, which is mainly secondary to the leakage of electrons from complex I of the respiratory chain to oxygen [23,24], and their impaired elimination by mitochondrial antioxidant system [20,25]. In fact, mitochondrial hydrogen peroxide (H2O2) detoxification is mediated by glutathione peroxidase and the peroxiredoxin/thioredoxin system, which are regenerated in their active form by a series of redox reactions ultimately requiring nicotinamide adenine dinucleotide phosphate (NADPH) as an electron acceptor [26]. In turn, the mitochondrial NADPH pool is maintained by the activity of the Krebs cycle, which produces isocitrate and NADH required for NADPH regeneration by the isocitrate dehydrogenase 2 (IDH2) and the nicotinamide nucleotide transhydrogenase (NNT), respectively. Therefore, impaired mitochondrial Ca2+ accumulation hinders both energetic adaptation and ROS elimination in mitochondria by blunting the Krebs cycle-dependent regeneration of NADH and NADPH [27].

Key alterations of cytosolic Ca2+ and Na+ handling have been observed in cardiac myocytes of patients with both ischemic and non-ischemic forms of cardiomyopathy [18], the latter typically determined by genetic mutations [28,29]. Based upon this, it may be assumed that the long-term consequences of hereditary forms of dilated cardiomyopathy (DCM) also eventually result in the above described alterations of EC coupling and mitochondrial redox regulation [6], even if the genetic defects do not affect mitochondria or metabolism per se. Similarly, secondary changes in substrate utilization, and mitochondrial structure and function may occur in HFrEF [5].

## 3. Dilated Cardiomyopathy

DCM is a common cause of HF and cardiac transplantation in young adults, with an estimated prevalence of 1:2500 and up to 1:250–400 [30,31,32], and is characterized by ventricular dilatation and contractile dysfunction. While it is clear that DCM can be determined by both genetic and non-genetic factors, the mechanisms underlying its development remain poorly understood. The majority of DCM mutations involve genes encoding for structural proteins of the cardiac cytoskeleton, sarcomere, or nuclear envelope [32,33,34] and their direct link with mitochondrial metabolism has been marginally investigated. On the other hand, experimental alterations of mitochondrial and metabolic genes induced not only defects in EC coupling and redox regulation, but also DCM features, although they may not represent the primary cause of the disease and the obtained data were not evaluated in humans. Below we report the studies for both categories.

### 3.1. Human DCM Mutations Affecting Mitochondria

Only very few studies linked human DCM mutations to mitochondrial abnormalities so far. Truncating mutations in the gene encoding the sarcomeric protein titin (TTN), frequently found in DCM patients [35], were associated with increased risk of ventricular arrhythmias, decreased cardiac mass as well as significant transcriptional upregulation of all components of the mitochondrial oxidative phosphorylation (OXPHOS) system [36]. The increased levels of respiratory chain complexes could reflect an attempt to enhance ATP production to compensate for the sarcomeric defects related to TTN mutation. More recently, TTN-truncating variants leading to DCM were studied in rats and correlated with impaired autophagy, decreased O2 consumption rate, excessive ROS production, and increase of mitochondrial protein ubiquitination in cardiomyocytes [37]. Additionally, aberrant ERK1/2 signaling was associated with altered mitochondrial shape, distribution, fragmentation, and degeneration in a mouse model of DCM carrying the p.H222P mutation in lamin A/C (*LMNA*) gene [38].

### 3.2. Perturbation of Mitochondrial Genes Recapitulating DCM

Although mutations in metabolic genes have never been reported as the direct cause of DCM in humans, several genetically engineered models for these genes recapitulated also DCM features. These studies did not prove the direct link between metabolic alterations and DCM, however they could represent a valuable starting point to analyze the metabolic remodeling in DCM. The work by Li and colleagues [39] investigated the role of ROS production and antioxidant defenses in mice lacking the mitochondrial manganese-superoxide dismutase (SOD2) gene. Strikingly, these mice exhibit the typical DCM phenotype. When a free radical scavenger was administered to SOD2-deficient mice, ventricular dilatation was reversed and the progression of cardiac dysfunction halted. Recently, Zhang et al. showed that mitochondrial transcription factor A (Tfam) inactivation in neonatal mice resulted in DCM, OXPHOS defect and elevated mitochondrial ROS production, which in turn led to DNA damage and cell-cycle arrest [40]. Accordingly, ROS scavenging ameliorated the detrimental effect of Tfam inactivation in this model, suggesting that exaggerated ROS production may play a crucial role in the pathogenesis of DCM. The link between OXPHOS alteration and impaired Ca2+ handling in the context of DCM was further elucidated by a mouse model of cardiac-specific deletion of the estrogen-related receptor-β (ESRRβ) [41]. Genetic deletion of ESRRβ led to the development of DCM, reduced expression of OXPHOS genes in adult cardiomyocytes, increased Ca2+ sensitivity and impaired contractility. Aberrant Ca2+ handling preceded cardiac remodeling and dysfunction, suggesting a potential role for impaired Ca2+ balance in the pathogenesis of DCM. Moreover, since many of the key proteins involved in intracellular Ca2+ handling are ATPases, OXPHOS defects may cause aberrant Ca2+ fluxes in the myocardium.

Other studies on animal models indicate that the modulation of the expression of genes involved in FA metabolism [42,43,44,45], mitochondrial dynamics [46,47] and integrity [48] can induce also DCM features, although it is not clear whether the same mechanisms occur also in human patients.

Overall, only a minority of the studies performed so far investigated the metabolic consequences of specific DCM mutations found in human patients. The evaluation of mitochondrial function and morphology in models harboring mutations causing DCM in humans could help elucidating the real pathogenic events occurring in the disease. Moreover, although the models in these studies recapitulate DCM features, further effort is required to understand whether the described mechanisms, resulting from genetic modifications in genes unrelated to the disease, occur also in DCM patients. On the other hand, despite the lack of direct evidences of metabolic alterations in models carrying specific DCM mutations, several mitochondrial diseases, such as Barth syndrome [49,50,51,52], OXPHOS disorders [53,54,55,56], and Leigh syndrome [57,58], are associated with the occurrence of DCM, suggesting a role of mitochondrial alterations in the pathogenesis of DCM.

## 4. Hypertrophic Cardiomyopathy

Hypertrophic cardiomyopathy (HCM) is the most common inherited form of HF (1:200 prevalence) and is mainly caused by mutations of genes encoding sarcomeric proteins that increase the Ca2+ affinity of the myofilaments [59,60]. The most relevant clinical consequences are diastolic dysfunction and arrhythmias, with HCM being the most common cause of sudden cardiac death in young individuals [61,62]. The dominant macroscopic feature of HCM is the extensive hypertrophy of the left ventricle (LV), which involves predominantly the interventricular septum. Most patients display asymmetric septal hypertrophy in combination with LV outflow tract obstruction at rest or during exercise. Only a small minority of individuals (–5%) ultimately develop systolic HF, characterized by reduced ejection fraction (<50%), cavity dilation, and regression of hypertrophy, i.e., dilated-hypokinetic (end-stage) evolution of HCM [63].

HCM patients can be severely symptomatic, showing the typical myocardial hypertrophy accompanied by cellular disarray, fibrosis and microvascular dysfunction, or remain asymptomatic, with a structurally normal heart and microvascular function [61,62]. Myocardial ischemia is commonly reported in symptomatic HCM subjects who survived cardiac arrest [64], which might represent the direct consequence of reduced cardiac perfusion [65,66]. In this context, the high metabolic demand of the hypertrophic myocardium is not matched by an adequate vascular supply. This is more frequently observed in HCM with symptomatic LV outflow obstruction, where the myocardium faces an additional energetic burden imposed by the elevated afterload resembled by left ventricular outflow tract obstruction [67].

Several studies on patients with HCM support the concept that in the hypercontractile myocardium, inadequate creatine kinase-phosphocreatine coupling results in insufficient ATP regeneration and subsequent diastolic dysfunction [68]. A more recent study from Güçlü and coworkers [69] shows a reduction of myocardial external efficiency (the ratio of external work to myocardial oxygen consumption) in both asymptomatic (28% of cardiac efficiency (low cardiac work but higher O2/g consumption)) and symptomatic (22% of cardiac efficiency (high cardiac work but lower O2/g consumption)) HCM patients compared to controls (42% of myocardial external efficiency). Notably, myocardial O2 consumption was higher in asymptomatic HCM patients compared to control individuals, while it was greatly diminished in symptomatic HCM [69]. It is unclear from the study whether the reduced O2 consumption in symptomatic HCM results primarily from microvascular dysfunction (as previous reports show in manifested HCM [65,66]) or the metabolic shift towards glucose oxidation that reduces oxygen utilization. Based on the findings from Güçlü et al. [69], one can estimate that symptomatic HCM patients have higher metabolic requirements compared with asymptomatic ones, with a shift towards glucose oxidation at the expense of increased septal mass and elevated outflow gradient pressures. Accordingly, these data are corroborated by a recent study from Aoyama and colleagues [70], where nuclear imaging indicates extensive glucose uptake in the symptomatic HCM but not in the asymptomatic form of HCM without cardiac hypertrophy.

While in HCM, the initial cause is frequently a mutation of genes encoding sarcomeres, in the course of the disease, an additional hemodynamic burden may be imposed on the myocardium if left ventricular outflow tract obstruction occurs (hypertrophic obstructive cardiomyopathy). In this case, an additional trigger for hypertrophy is set by the afterload increase, which has been recapitulated in a number of experimental studies with thoraco-aortic constriction (TAC). From these studies, a vast body of evidence investigated metabolic remodeling in TAC-induced hypertrophy and HF, and the majority of these studies observed a shift away from FA and toward glucose oxidation for ATP production. FA metabolism depends on the tight balance between their synthesis, uptake and oxidation. FAs differ in size (carbon chain length), degree of saturation (number of double bounds), and geometry (cis or trans). For instance, long- and very-long chain FAs (>16 carbon atoms) require FA transporters to be internalized in cardiomyocytes, the most relevant being cluster differentiation 36 (CD36). However, CD36 expression is reduced in HF models [5]. Considering that the major FAs used by the heart are saturated long-chain FAs with 16 and 18 carbons atoms (palmitic and stearic acid, respectively) [71], this can partly explain the decrease in FAO encountered in HF. Similarly, CD36 expression is reduced in HCM animal models with manifested hypertrophy, suggesting impaired uptake of FA [72]. It is interesting to note that a previous study in human HCM detected CD36 deficiency in symptomatic patients, while asymptomatic HCM (i.e., without cardiac hypertrophy and HCM-related mutations) had normal CD36 levels [73], suggesting that changes in CD36 availability associate with the metabolic shift of FAs towards glucose consumption. This is in agreement with the abovementioned study of Güçlü and coworkers [69] showing that asymptomatic HCM patients have a very high oxygen demand (consistent with a shift toward FAO), which is substantially reduced upon the development of cardiac hypertrophy, potentially reflecting a shift toward enhanced glucose utilization. The study from Aoyama and colleagues [70] further validates this idea, because glucose uptake was mostly observed in symptomatic manifested HCM, but not in asymptomatic HCM carriers.

In spontaneous hypertensive rats with LV hypertrophy, which is associated with reduced CD36 and lower FAO, supplementation with caprylic acid, a medium-chain FA that bypasses FA transporters, reduced ventricular hypertrophy and fibrosis, improved myocardial energetics (as measured by phosphocreatine levels) and reduced oxidative stress markers [74]. Recently, three independent studies [75,76,77] proposed that ketone oxidation might become a dominant source of ATP in the failing heart. Ketone molecules are produced from acetyl-CoA in the liver and subsequently released into circulation to provide extrahepatic tissues with a substrate for energy regeneration during conditions of carbohydrate depletion (e.g., prolonged fasting or starvation). Ketones are four carbon atoms in size. In a manner similar to medium-chain FA, ketone molecules bypass FA transporters, which may provide further indication that the failing heart may be partly “forced” to shift its fuel source away from long-chain FAO due to maladaptive remodeling that reduce CD36 availability. Currently there are no studies and/or targeted therapies using ketones or medium-chain FA as alternative fuels in HCM.

The metabolic treatment of symptomatic HCM patients is in its infancy. Perhexiline, a metabolic drug that shifts substrate utilization from FA towards glucose, induced modest improvements of the exercise capacity in symptomatic HCM patients [78] and reduced cardiac mass in a mouse model of HCM [79]. However, one must question whether the positive effects of perhexiline in symptomatic HCM are indeed caused by a shift from FA to glucose utilization or are mediated by the pleiotropic effects of the drug [80]. In fact, perhexiline also rebalances carbon and nucleotide fluxes via increases of lactate and amino acid uptake to maintain cardiac output [81], but may additionally reduce oxidative stress [79,82,83]. Treatment with ROS scavengers (e.g., N-acetylcysteine, NAC) in HCM models show positive effects on cardiac function, accompanied by the reduction of ventricular hypertrophy, fibrosis, and myocyte disarray [84,85,86]. Accordingly, in symptomatic HCM patients, lower catalase activity is observed, while the levels of other antioxidant enzymes, including mitochondrial superoxide dismutase (SOD) and glutathione peroxidase (GPX), are unaltered [87]. Similar findings were reported in a pig model of naturally occurring HCM [88]. Notably, elevated oxidative stress is an established driver of left ventricular hypertrophy and fibrosis in HCM models [84,85,86,89,90,91], which suggests that this event precedes the progression of the disease from the asymptomatic to the symptomatic form.

In summary, metabolic remodeling follows the progression of cardiac hypertrophy in human HCM and is accompanied by reduction of CD36, decreased dependency on FAO for ATP production, higher myocardial glucose uptake, and lower oxygen consumption. Evidence supporting the use of metabolic agents for the treatment of HCM is currently limited, but the benefits of drugs acting on substrate preference may largely depend on the stage of disease. For instance, based on the observation that CD36 levels decrease in the transition from asymptomatic to symptomatic HCM, it appears counter-productive to use metabolic drugs in asymptomatic HCM patients. On the contrary, the use of medium-chain FA and ketone supplementation to bypass the reduction in CD36 in symptomatic HCM might be potentially more beneficial. Finally, oxidative stress markers appear to increase with the progression of manifested HCM, which can partly explain the development of myocardial hypertrophy and fibrosis.

### 4.1. Human HCM Sarcomeric Mutations Directly Affecting Mitochondria

High levels of ROS are observed in HCM [85,86,89,90,91]. In accord, HCM patients exhibit an increase in oxidative stress markers via lipid, DNA, and protein damage in the heart [91,92,93] and serum [90], with a trend towards higher levels in the symptomatic form of the disease [90,91]. HCM sarcomeric mutations are likely responsible for such large increases in ROS production in mitochondria. As mentioned above, during elevations of cardiac workload, increases of cytosolic Ca2+ dynamics enhances the rate of ATP consumption, thereby accelerating mitochondrial ADP-workload. At this point, Ca2+ accumulates in the mitochondrial matrix and stimulates Krebs cycle dehydrogenases to regenerate NADH, as to match increases in ATP demand leading to high ATP regeneration at the mitochondrial respiratory chain. Because HCM sarcomeric mutations enhance myofilament Ca2+ affinity and diastolic stress/workload [94,95], the ATP demand for any given cytosolic Ca2+ level is higher in HCM myocardium. We previously observed, in a mouse model of pressure overload, that the increased cardiac workload causes the mismatch between high NADH oxidation and inadequate mitochondrial matrix Ca2+ dependent stimulation of the Krebs cycle [96]. Notably, ATP regeneration was however prioritized at the expense of mitochondrial anti-oxidative capacity, because NADH oxidation reversed the reaction of the mitochondrial NNT, thereby consuming NADPH (essential to NADPH-linked anti-oxidative capacity) to support NADH and ATP production [96]. Having in mind that in human HCM the mutations are expected to increase cardiac and mitochondrial ADP-workload [94,97], one can speculate a similar energetic mismatch to support ATP regeneration at the cost of adequate anti-oxidative capacity. The resulting mitochondrial overflow of H2O2 produces oxidative stress [96], likely accounting for the high levels of ROS observed in HCM [85,86,89,90,91].

Localized ROS at the mitochondria damages mitochondrial proteins, but also oxidative damage to mitochondrial DNA, which tends to increase in HCM [98], affects translation of several proteins involved in the assembly of mitochondrial complexes I, III, IV, and V from mitochondrial DNA [99] (Figure 2). Along these lines, oxidative stress damages mitochondrial membrane lipids (but also other membrane lipids), causing the formation of lipid radical and peroxides [100] that affect membrane fluidity and structure. In HCM, markers of lipid peroxidation are frequently found elevated [91,98,101]. Lipid peroxidation to mitochondrial membranes destabilizes mitochondrial membrane integrity via disruption of the mitochondrial respiratory chain. In particular, a unique type of phospholipid, called cardiolipin (CL), is exclusively found, in mammals, in the inner mitochondrial membrane (up to 20%) with minor expression at the outer mitochondrial membrane (~3%) [102,103,104]. CL is necessary to provide structural and functional support to the respiratory chain complexes as it facilitates improvements of electron transfer flow and ATP regeneration, while simultaneous reducing electron leakage and ROS formation [50] (Figure 2). Nevertheless, because of its high (poly)unsaturated fatty acid nature (e.g., linoleic acid (18:2) [51]) CL is highly vulnerable to ROS, which disrupts respiratory chain complexes (i.e., the electron transport chain, ETC), but also depletes lipid-soluble compounds, including cytochrome c and coenzyme Q10 (CoQ10) [105]. Cytochrome c release from the mitochondrial membrane compartments activates cellular death [106], and CoQ10 administration in human HCM associated with reductions of outflow pressure gradient, improved diastolic dysfunction, ameliorated symptoms of dyspnea and fatigue, in addition to reductions of septal hypertrophy [107,108]. In our mouse model of pressure overload we tested the efficacy of drugs that restore mitochondrial bioenergetics, while reducing mitochondrial ROS production, as a potential therapeutic for heart failure patients. Perhaps the most important, the Szeto–Schiller peptide (SS-31, Elamipretide), a tetrapeptide currently under clinical investigation for non-genetic forms of systolic heart failure (NCT02788747), rescued mortality in pressure overload-induced failure animals [96]. We speculate that the positive effects of SS-31 are likely accounted by its role in the stabilization of cardiolipin in the inner mitochondrial and the mitochondrial complexes to improve ATP regeneration and reduce ROS formation [105]. SS-31 offers substantial therapeutic value for HCM patients.

### 4.2. Perturbation of Mitochondrial Genes Recapitulating HCM

In human HCM, genetic mutations are not limited to the sarcomeric gene but have been recently identified in mitochondrial DNA and mitochondrial-related nuclear DNA genes [109]. Identified mitochondrial DNA mutations in HCM patients involves genes that encode proteins of the mitochondrial ETC-complex I (MT-ND2 and MT-ND4, NADH dehydrogenases) and complex III (MT-CYB, cytochrome b), mitochondrial transfer RNA (MT-TG and MT-TS2) and mitochondrial ribosomal RNA (MT-RNR1 and MT-RNR2) [109]. At present, there is a lack of information regarding their contribution and prevalence to overall HCM. Mitochondrial-related nuclear DNA mutations identified in HCM are vaster and can be identified in proteins of the five mitochondrial complexes of the respiratory-chain genes, including mitochondrial assembly proteins. Because mitochondrial disorders are often multisystemic, and can mimic to some extent an HCM (or even DCM) clinical phenotype, these are separately discussed below.

## 5. Mitochondrial Disorders

Mitochondrial disorders are caused by hereditary or sporadic defects of mitochondrial energy metabolism often resulting in multisystemic syndromes with prominent cardiac involvement. In this context, disorders of the oxidative phosphorylation system (OXPHOSDs) are a heterogeneous group of diseases determined by mutations affecting the mitochondrial ETC and ATP synthase. Altogether, their prevalence is higher than 1 in 5000 in adults [110]. The OXPHOS consists of five multimeric enzyme complexes: complexes I-IV form the ETC, while complex V (F1Fo ATP synthase) catalyzes ADP phosphorylation (Figure 2). Additionally, the system includes two mobile electron carriers, CoQ10 and cytochrome c. Within the ETC, molecular oxygen is consumed and ATP is produced to maintain an adequate high ATP/ADP ratio for cardiomyocytes. Mutations in *NDUFS2* and *NDUFV2* genes, encoding for complex I subunits, and in *ACAD9* and *NDUFAF1* genes, encoding for assembly factors, have been associated with HCM as part of a multisystemic disease [111,112]. Additionally, mutations in the genes coding for the succinate dehydrogenase (complex II) subunits A (SDHA) and D (SDHD) lead to syndromic phenotypes, associated not only with HCM, but also with DCM and left ventricular non-compaction cardiomyopathy [53,54,55]. One of the least common OXPHOS defects is complex III deficiency. Patients carrying different mutations in the *MTCYB* gene, encoding for cytochrome b, showed HCM or histiocytoid cardiomyopathy [113,114,115,116]. Moreover, cardiac manifestations may occur in case of defects in complex IV, also known as cytochrome c oxidase (COX). Mutations in COX subunits genes (*COX6B1, MT-CO1, MT-CO2, MT-CO3*) and in COX assembly factors genes (*COA5, COX10, SCO1, SCO2, SURF1*) were reported in patients with multisystemic disease presenting DCM and HCM [56,117,118,119,120,121]. Mutations in *ATP6* and *ATP8* genes, encoding for complex V subunits, as well as in *TMEM70*, encoding a protein involved in the insertion of the ATP synthase into the mitochondrial membrane [122], lead to HCM both alone or in combination with other multisystemic defects. Several cases of CoQ10 deficiency associated with cardiomyopathy have been described in literature. In this case, hypertrophy is the main cardiac phenotype observed in patients carrying mutations in *CQQ2, CQQ4*, and *CQQ9* genes required for CoQ10 biosynthesis [123,124,125].

Another syndromic disease associated with mutations in the five OXPHOS complexes and with defective mitochondrial energy generation is Leigh syndrome [57,126,127,128,129,130], the most common progressive neurological infantile disorder, affecting 1 in 40,000 live births [58]. Patients with Leigh syndrome usually present developmental delay, hypotonia, ataxia, and ophthalmologic abnormalities, although this multisystemic disorder can also affect other organs, such as heart, liver, kidneys, and the gastrointestinal tract. Particularly, the most common cardiac manifestation in Leigh syndrome is HCM, while DCM has been rarely reported [58,127].

Friedreich’s Ataxia (FRDA) is a rare multisystemic disorder caused by homozygous GAA triplet repeat expansion in the FXN, encoding for the mitochondrial protein frataxin (FXN) [131]. Despite FRDA is mainly a neurological disorder, the affected patients may experience also HCM before the age of 40, which represents the major cause of death in these subjects [132,133]. In vitro studies on cells from affected patients suggest the implication of oxidative stress in the pathogenesis of FRDA, leading to increased mitochondrial iron [134,135]. These data are corroborated by a study on a conditional Fxn knockout mouse reporting the increased oxidative stress in the disease, following impaired Fe-S cluster synthesis [136]. In a recent paper from the group of Payne, lysine-acetylation of metabolic proteins was found increased in failing hearts in a Fxn knockout mouse model [137]. Interestingly, the acetylation pattern negatively correlated with the progression of cardiac function: as it increases, fractional shortening and ejection fraction decrease. Additionally, mitochondria resulted damaged in terms of collapsed cristae, disordered mitochondria-to-sarcomere arrangement, and compromised respiratory function. These results suggest early acetylation as a possible therapeutic target to halt or slow the progression of FRDA in the heart.

The impact of mitochondrial dysfunction on heart physiology is also exemplified by Barth syndrome (BTHS). Patients with BTHS present with cardiomyopathy, skeletal myopathy, neutropenia, and growth retardation [138]. BTHS cardiomyopathy is described as dilated or hypertrophic, in some cases associated with left ventricular non-compaction [49,50,51,52]. BTHS is caused by an inherited defect in the biogenesis of the phospholipid CL. As above mentioned, CL provides structural and functional support to several of the protein complexes residing in the inner mitochondrial membrane, including the respiratory chain complexes [50]. CL is a phospholipid with a glycerol head group esterified with two phosphatidylglyceride backbone molecules. In the mammalian heart, linoleic acid (18:2) is the predominant form of the four acyl chains bound to CL [51]. As other tissues show a greater variability in CL species, it has been proposed that the high energy demand of the heart requires a specific FA composition of CL [52,139]. CL deficiency may also be associated with another inherited cardiac disease, termed DCM with Ataxia (DCMA) [140]. Moreover, the CL amounts, species distribution, and peroxidation are altered in the diabetic, failing and aging heart and after ischemia and reperfusion injury [141,142,143,144,145,146]. CL is synthesized in the inner mitochondrial membrane. After its initial biosynthesis, CL is remodeled by an exchange of acyl chains by the subsequent action of the Ca2+-independent phospholipase iPLA2γ and the evolutionarily conserved enzyme tafazzin. BTHS is caused by loss-of-function mutations in the tafazzin gene, which results in a decrease in mature CL species and an accumulation of its precursor monolysocardiolipin (MLCL). The increased MLCL/CL ratio is used as a diagnostic marker for BTHS [147].

Different cell and animal models including yeast, lymphoblasts, *Drosophila*, *Caenorhabditis elegans*, *Trypanosoma*, mouse, and induced stem cell (iPSC)-derived cardiomyocytes have been designed to study the impact of CL deficiency on cellular physiology [148,149,150,151,152,153,154,155]. The defect in CL biogenesis in BTHS causes structural and functional changes in mitochondria that have a profound impact on cellular metabolism and physiology [149,156,157]. The morphological defects observed in BTHS mitochondria are explained by the interaction of CL with regulatory factors shaping mitochondrial morphology, like mitofusin 1 and 2, Drp1 and Opa1 [158,159,160,161,162], or with structural elements, forming mitochondrial cristae structures, like the mitochondrial contact site and cristae organizing system (MICOS) [163,164,165]. Moreover, mitochondria are organelles of dual genetic origin with a limited set of proteins encoded by mitochondrial DNA. The translation of mitochondrial genes is severely affected in CL-deficient cells. The majority of mitochondrial proteins are encoded in the nucleus and are imported by protein translocases in the outer (translocase of outer membrane, TOM) and inner membrane (translocase of inner membrane, TIM23), which were found to be structurally dependent on CL [50]. In summary, CL plays an important role in mitochondrial biogenesis and morphology.

The role of CL in mitochondrial metabolism is exemplified by its involvement in oxidative phosphorylation. CL is an integral constituent of all five complexes of the respiratory chain and the ADP/ATP carrier [166,167,168]. The structural and functional support provided by CL to protein complexes is particularly important for complex III, where CL plays a role in dimer formation [169]. Furthermore, complex IV subunits, which were found in close proximity to CL in the crystal structure, dissociate under conditions of CL deficiency, indicating that CL plays a role in the structural integrity of the complex [170]. Interestingly, one of these CL-dependent subunits (COX VIa) is expressed as a cardiac-specific isoform and has an important regulatory function, providing a possible explanation for the cardiac-specific phenotype in BTHS patients. Besides the structural function, CL is required for the enzymatic activity of complex II, where one CL molecule is located near the binding site for ubiquinone, suggesting that CL is involved in the proton pumping mechanism. A role for CL in proton transport has been recently proposed also for complex IV [171]. In cellular models of BTHS, including patient-derived iPSC, oxygen consumption at the respiratory chain is reduced, reflecting the impaired activity of individual respiratory chain complexes and diminished energetic coupling between complexes [148,154,156].

Complex I, dimeric complex III, and complex IV are organized into large supercomplexes, termed respirasomes [172,173]. Cardiac mitochondria from the BTHS mouse model and patient-derived iPSC cardiomyocytes display a dramatic reorganization of respirasomes [174,175]. Respirasome organization was suggested to guarantee efficient energy coupling between respiratory chain complexes, and in fact an increase in the generation of ROS was measured in numerous cellular models of BTHS [176]. The electron carrier cytochrome c in the respiratory chain might also be a dominant source of ROS under conditions of CL deficiency. Alteration in the direct interaction of CL with cytochrome c leads to a conformational change, which induces a peroxidase activity of cytochrome c, consequently producing large amounts of ROS [177]. These data provide insights into how defects in lipid biogenesis affect the structure and function of the ETC and how these defects lead to cardiomyopathy. Of note, also in a large animal model of HF, defects in the assembly of respirasomes have been observed [178].

The oxidative stress in BTHS resulting from the structural and functional changes in the respiratory chain may have severe implications for cardiac function. Studies on iPSC-derived cardiomyocytes indicated a severe defect in the assembly of sarcomeres and a consequent decrease in contractile force in BTHS patient cells. This defect was ameliorated upon treatment with antioxidants [155]. A recent study links oxidative stress to abnormalities in Ca2+ handling in the BTHS mouse model. The SR Ca2+-ATPase (SERCA) is responsible for the Ca2+ reuptake into the SR during diastole. Oxidative stress induces SERCA nitrosylation in BTHS, which results in a lower SERCA activity and a decrease of SR Ca2+ content [179].

The majority of BTHS patients remain stable on standard HF medical therapy. Therapeutic approaches targeting mitochondrial CL deficiency and the resulting metabolic changes are currently emerging. In an experimental approach, gene replacement therapy of the defective tafazzin gene was successfully tested in cells and in the BTHS mouse model [180,181]. Following the concept of directly targeting mitochondrial CL, SS-31was tested in different experimental and clinical approaches. This mitochondria-targeted tetrapeptide was shown to directly interact with CL and prevent cytochrome c peroxidase activity in many experimental settings. However, it was not beneficial in a phase II clinical trial of 12 BTHS patients (TAZPOWER trial, NCT03098797). Furthermore, a metabolic approach to the treatment of BTHS was recently addressed using bezafibrate, an agonist of the transcription factor peroxisome proliferator-activated receptor (PPAR). PPAR regulates a large number of genes involved in energy conversion and FA metabolism. Studies in mice indicate a benefit in cardiac function and in exercise capacity in BTHS mice treated with bezafibrate [182,183]

In summary, CL deficiency in BTHS has severe impact on many mitochondrial functions, hindering ATP production and inducing oxidative stress. Moreover, mitochondria are also a central hub for cellular signaling pathways [184]. Recent data indicate an impact of CL deficiency on key cellular signaling pathways involved in the response to hypoxia [185]. BTHS cell and animal models have revealed the impact of dysfunctional mitochondria on cardiac function. A better understanding of the molecular mechanism involved in BTHS will lead to new concepts in the treatment of BTHS.

Sengers syndrome, also known as cardiomyopathic mitochondrial DNA depletion syndrome-10 (MTDPS10), is another mitochondrial multisystemic disorder that presents HCM as the dominant clinical manifestation, together with congenital cataract, muscle weakness, and lactic acidosis [186]. Although the pathological mechanisms are unclear, defects in acylglycerol kinase (AGK) and, downstream, in the synthesis of CL, are reputed to play a key role in the disease [140,187]. It is currently known that CL directly interacts with OXPHOS components and that it is required for stability, integrity, and full activity of all the five complexes of ETC [188]. Accordingly, disruption of the CL pool can negatively impact the activity and efficiency of OXPHOS. However, two recent studies showed that AGK may be involved in the pathogenesis of Sengers syndrome via mechanisms unrelated to lipid metabolism. AGK is a subunit of the mitochondrial TIM22 complex, which mediates the insertion of metabolite carriers into the inner membrane [189,190]. Destabilization of the TIM22 complex and protein import defects were observed by Kang et al. in mitochondria isolated from patients with Sengers syndrome [190]. Moreover, in HEK293T cells with permanent AGK knock-out, the authors found reduced mitochondrial respiration, ETC complexes instability, and metabolic reprogramming promoting perturbations in the tricarboxylic acid cycle [189]. These findings revealed that both defects in lipid biosynthesis and in mitochondrial protein import via TIM22 complex might contribute to the pathogenesis of Sengers syndrome.

Although both nuclear and mitochondrial DNA (mtDNA) encode for proteins involved in mitochondrial biology, only point mutations in the latter are responsible for the majority of mitochondrial diseases. MtDNA encodes 13 essential proteins, including OXPHOS components and essential translational machinery elements required for their synthesis, such as mt-rRNAs and mt-tRNAs. In particular, a significant number of mt-tRNA gene mutations occurs into three mt-tRNAs, tRNAIle, tRNALeu, and tRNALys, being responsible for a broad variety of mitochondrial disorders, including the so-called mitochondrial myopathy, encephalopathy, lactic acidosis, and stroke-like episodes (MELAS) multisystemic syndrome [191,192]. More than 80% of MELAS patients present mutations in the mitochondrial tRNALeu gene (MT-TL1) and approximately a third of MELAS patients develops cardiomyopathy [193,194,195]. Myoclonic epilepsy with ragged red fibers (MERFF) is another multisystemic disorder associated with mt-tRNA mutations [196]. In this case, around 80% of cases is caused by alterations in the tRNALys, encoded by the MT-TK gene. Myoclonus, epilepsy, ataxia, muscle weakness, and cardiomyopathy have been reported in MERFF [196]. In general, it is currently assumed that patients with MELAS and MERFF should be carefully monitored for the development of cardiac hypertrophy and DCM [197].

Although they have been mostly associated with multisystemic diseases, mt-tRNA defects have been described also linked to perturbations involving exclusively the cardiac muscle. For instance, Giordano and colleagues observed mitochondrial cardiomyopathy in three transplanted patients carrying mutations in the MT-TI gene, coding for mitochondrial tRNAIle [198]. The analyzed hearts showed symmetric hypertrophy and hypertrophic cardiomyocytes revealed a respiratory chain defect mainly due to lack of COX.

## 6. Fatty Acid Oxidation Disorders

FA oxidation disorders (FAODs) include defects of metabolism caused by abnormalities in either mitochondrial β-oxidation or the import of FA in the mitochondrial matrix via the carnitine shuttle [199]. The former are determined by recessive mutations in genes encoding for the very long-chain acyl-CoA dehydrogenase (VLCAD), the long chain 3-hydroxy-acyl-CoA dehydrogenase (LCHAD) and the trifunctional mitochondrial protein (TFP). In particular, decreased levels of FAO enzymes catalyzing the first steps in β-oxidation of long chain FA have been associated with fatal cardiomyopathy in young patients. Similar to what is observed in mouse models showing deficiencies in FAO enzymes, humans with comparable metabolic defects exhibit cardiac steatosis and a wide spectrum of cardiomyopathies, including HCM [200,201,202,203].

On the other hand, the long-chain FA transport system is based on carnitine. In this three-step process, long-chain FA are conjugated to carnitine via the CPT1 transferase, transported across the mitochondrial inner membrane by the carnitine-acylcarnitine carrier (CAC), and released as acyl-CoA by the CPT2 transferase, to be used for β-oxidation [204,205]. While mutations in the CPT1 transferase genes have not been associated with cardiac malfunctions, recessive mutations in the CAC gene, SCL25A20, have been associated with multisystemic disorders involving also heart defects, such as mild hypertrophic cardiomyopathy, arrhythmias, and cardiac insufficiency as a consequence of the marked reduction in FAO [206]. Similarly, the deficiency in CPT2 was also associated with arrhythmias and cardiomyopathy [207].

It is currently widely known the central role of PPARγ coactivator (PGC)-1α in mitochondrial biogenesis and function, particularly as regulator of gene expression from both nuclear and mitochondrial genomes. PGC-1α is highly expressed in tissues with a high energy demand, such as brown adipose tissue and heart [208]. Accordingly, decreased expression of PGC-1α has been observed in several models of heart failure, suggesting its contribution to the maladaptive energy response in failing hearts [209]. In a study performed by Sano et al., the suppressed expression of PGC-1α by cyclin-dependent kinase 9 promoted cardiomyopathy [210]. Moreover, a report in mice showed that PPARγ and PGC-1α mRNA levels were reduced in pressure overload-induced cardiac hypertrophy compared to controls, together with the downregulation of FAO enzyme genes [211]. While the decreased expression of PGC-1α in the heart is associated with FAO alterations, on the other hand, PGC-1α cardiac-specific overexpression induced uncontrolled mitochondrial proliferation and cardiomyopathy in two independent transgenic mouse models [212,213]. In particular, the work of Lehman et al. described loss of sarcomeric structure and dilated cardiomyopathy [212], while increased ventricular mass and chamber dilatation was found by Russell et al. after PGC-1α overexpression [213]. Overall, abnormal PGC-1α function is likely to play a pivotal role in the pathogenesis of metabolic cardiomyopathies.

## 7. Storage Disorders

Storage disorders represent one of the most frequent classes of inborn errors of metabolism (IEMs) consisting in the accumulation of metabolites caused by the disruption of their breakdown process. These diseases commonly present cardiac manifestations, mostly ventricular hypertrophy progressing to dilation, and are classified according to the enzymatic deficiency involved in the process [58].

The protein kinase AMP-activated non-catalytic subunit gamma 2 (PRKAG2) cardiac syndrome is an autosomal dominant metabolic heart disease characterized by cardiac hypertrophy and conduction abnormalities. In this storage disorder, PRKAG2 mutations promote abnormal PRKAG2-mediated activity of the AMP-activated protein kinase (AMPK) within glycogen metabolism, with consequent substrate accumulation [214,215,216]. The molecular mechanisms underlying the cardiac manifestations are currently unknown, but they seem to correlate with abnormal glycogen storage in the myocardium [217]. This association was highlighted in a study showing that inhibition of glycogen accumulation in cardiomyocytes can suppress the conduction defects in transgenic mice overexpressing a PRKAG2 missense mutation and showing cardiac hypertrophy, ventricular pre-excitation, and sinus and atrioventricular node dysfunction [217]. More recently, Xu and colleagues reported that also the AMPK functions unrelated to glycogen metabolism might be compromised in PRKAG2 cardiac syndrome. In this study performed in rat H9c2 cardiomyocytes, the abnormal activation of the mTOR pathway, which is normally inhibited by AMPK, was indicated as a potential mechanism involved in HCM caused by PRKAG2 mutations. This hypothesis was corroborated by decreased hypertrophy in rat embryonic cardiomyocytes after mTOR pathway inhibition [218].

Also known as glycogen storage disease type II, Pompe disease is another storage disorder, leading to α-glucosidase (GAA) deficiency and subsequent intralysosomal build-up of glycogen in the affected tissues, including heart, skeletal muscle, and liver [219]. Pompe disease is inherited as an autosomal recessive trait and presents with a broad clinical spectrum that varies with respect to age and onset, rate of disease progression, and organ involvement [220]. Cardiac phenotypes include severe HCM or DCM with conduction abnormalities [221]. While the pathologic mechanisms of Pompe disease are not clear, more is known about Danon disease, an X-linked lysosomal disorder caused by mutations in the LAMP2 gene (lysosome-associated membrane protein 2) and associated with cardiomyopathies, including DCM and HCM [222,223]. Particularly, the first study performed on heart muscle from LAMP2-deficient mice showed accumulation of autophagic vesicles and glycogen deposits associated with reduced contractile function [224]. More recently, autophagy defects coupled with impaired mitochondrial clearance, also known as mitophagy, were reported by Hashem et al. in both human iPSC-derived cardiomyocytes carrying LAMP2 mutations and in LAMP2-deficient mice [225]. In these models, impaired mitochondrial respiratory capacity and abnormal gene expression of key mitochondrial pathways were observed, shedding light on pathological mechanisms of Danon disease, that might become potential targets for therapeutic intervention.

Fabry disease is a rare X-linked lysosomal storage disorder caused by a range of mutations in the gene encoding GLA, leading to α-galactosidase A deficiency [226]. It is characterized by accumulation of globotriaosylceramide (Gb3) in lysosomes within various tissues including the nervous system, kidneys, eyes, skin, and heart [227]. As a consequence of Gb3 accumulation, the activities of enzymes I, IV, and V of the respiratory chain are reduced, with subsequent reduction of levels of energy-rich phosphates [228]. This, together with the release of pro-inflammatory cytokines, growth-promoting factors, and oxidative stress are thought to compromise heart function. Myocardial extracellular matrix remodeling, left ventricular hypertrophy, systolic and diastolic dysfunction, valvular abnormalities, vascular dysfunction, and conduction tissue disease are common features in Fabry disease [229,230,231], resulting in a considerable cardiovascular morbidity and mortality as well as sudden cardiac death [232,233].

## 8. Diagnostic Aspects

**Figure 3:**
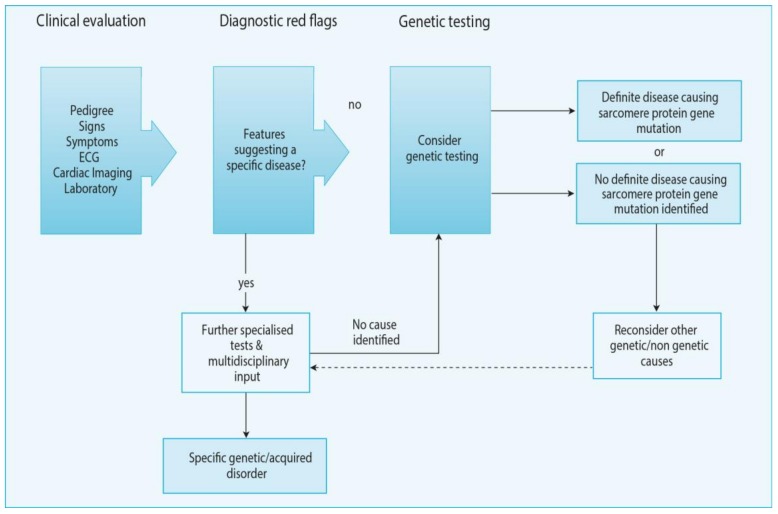
Schematics of the general approach to the diagnosis of HCM. ECG, electrocardiogram. Image adapted with permission European Society of Cardiology (ESC) Guidelines on Diagnosis and management of HCM [234].

The clinical diagnosis of HCM is based on the presence of left ventricular hypertrophy that is not explained solely by abnormal loading conditions. As discussed in detail above, there are multiple underlying causes of HCM, which have a symptomatic and diagnostic overlap with restrictive cardiomyopathy and which need to be differentiated by a systematic diagnostic work-up which is covered in detail in the current (2014) European Society of Cardiology (ESC) Guidelines on Diagnosis and management of HCM [234]. Furthermore, we refer the reader to in-depth reviews on the clinical differential diagnostics of hypertrophic and restrictive cardiomyopathies [235]. Furthermore, some of the clinical manifestations which can be considered “red flags” for the presence of hereditary forms of cardiomyopathy are listed in Table 1 and Table 2. In brief, when a patient presents with a suspected cardiomyopathy, a clinical evaluation that comprises the pedigree, signs and symptoms, ECG, cardiac imaging (echocardiography and/or cardiac magnetic resonance imaging) and laboratory testing (Figure 3). If any of the “red flags” listed in Table 1; Table 2 are observed, the patient should be referred to further specialized tests and multidisciplinary input in a dedicated center. There, genetic testing should be considered to identify a definite disease causing sarcomere protein gene mutation. If no such gene is identified, a search for other genetic or non-genetic causes should be considered. After the diagnosis is made, specific treatment as suggested by the current Guidelines is recommended [234].

## 9. Conclusions

In conclusion, inherited cardiomyopathies represent one of the most frequent causes of death and hospitalization worldwide, with a significant economical and societal impact. Understanding the underlying pathogenic mechanisms of these disorders and the development of novel therapies that could subclass the current palliative approaches is hence urgently needed. Despite the unique morphological disease-specific features, derangements in the cardiac energy metabolism machinery is a frequent feature in this class of disorders, involving substrate utilization, mitochondrial oxidative metabolism, and redox regulation. All these levels potentially represent a novel therapeutic target for the cardiomyopathies. In this context, clinical trials for certain phenotypes are currently open, but there is still a need for further studies to continue evaluating the current understanding and to implement future therapies. More benefit could also arise from studies on the genotype-phenotype correlation, which is still unclear, but could help to develop personalized therapeutic approaches as well as to stratify cardiopathic patients. Finally, patients could also benefit from an improved diagnosis of this class of diseases. In most metabolic cardiomyopathies, the exclusive heart involvement is a rare feature, while, most frequently, cardiac alterations can be an incidental or minor finding, discovered during multisystemic evaluation. This highlights the complexity of the diagnosis of these diseases, which is currently based upon clinical and familiar history and a physical and metabolic assessment (Figure 3). Further research in the development of these diseases could enhance diagnostic strategies and establish early therapies improving the patients’ quality of life and prognosis.

## Figures and Tables

**Table 1:** Alterations in cardiac metabolism in inherited cardiomyopathies.

Disease	Affected genes and proteins		Phenotype	Consequences on cardiac metabolism
Dilated cardiomyopathy (DCM)	Mutations of genes encoding proteins of the cardiac cytoskeleton (e.g., *DES*), sarcomere (e.g., *TTN*), or nuclear envelope (e.g., *LMNA*).		Ventricular dilatation, contractile dysfunctions, reduced systolic function. Clinical features: arrhythmias, heart failure, SCD.	↑Transcription of OXPHOS components ↑ATP production ↓Oxygen consumption ↑ROS Aberrant ERK1/2 signaling associated with altered mitochondrial properties.
Hypertrophic cardiomyopathy (HCM)	Mutations of genes encoding sarcomeric proteins (e.g., myosin heavy chain; myosin binding protein C). Mutations in mtDNA and mitochondrial-related nDNA genes.		Extensive hypertrophy of the left ventricle with interventricular septum involvement. Clinical features: arrhythmias, diastolic heart failure, SCD.	↓CD36 expression ↓Fatty Acid Oxidation ↑Ketone oxidation ↑Glucose oxidation ↓Oxygen consumption ↑ROS
Barth syndrome	Defects in Cardiolipin (CL) biogenesis. TAZ gene mutations.		Multisystemic disease. Cardiomyopathy (DCM, HCM, LVNC), skeletal myopathy, neutropenia, growth retardation.	Structural and functional changes in mitochondria ↓Translation of mDNA genes ↓Import of mitochondrial proteins encoded by nDNA ↓Activity of ETC ↓Oxygen consumption ↑ROS leading to: altered sarcomere assembly, ↓contractile force, SERCA functional abnormalities, ↓SR Ca^2+^ levels
OXPHOS disorders	CID	*ACAD9, NDUFAF1*	Multisystemic syndromes including cardiac defects (DCM, HCM, LVNC).	Altered ECT function leading to impaired mitochondrial oxidative phosphorylation.
	CIID	*SDHA, SDHD*		
	CIIID	*MTCYB*		
	CIVD	*COX6B1, MT-CO1/2/3, COA5, COX10, SCO1/2, SURF1*		
	CVD	*ATP6, ATP8, TMEM70*		
	CoQ10D	*CQQ2, CQQ4, CQQ9*		
Leigh syndrome	Mutations in all the genes encoding the 5 respiratory chain complexes. Mutations in pyruvate metabolism and fatty acid oxidation genes.		Multisystemic disease. Developmental delay, hypotonia, ataxia, ophthalmologic abnormalities. Heart can also be involved. HCM is the main cardiac manifestation.	Altered ECT function promoting defective mitochondrial energy production.
Friedreich’s Ataxia (FRDA)	Homozygous GAA triplet repeat expansion in the *FXN* gene		Multisystemic disease. Neurological disorders and cardiac manifestations (HCM).	Impaired mitochondrial iron metabolism Impaired mitochondrial respiratory function Mitochondria abnormalities. ↑ Oxidative stress ↑ lysine-acetylation of metabolic proteins
Sengers syndrome	*AGK*		Multisystemic disease. HCM is the dominant clinical manifestation. Cataract, muscle weakness, lactic acidosis.	AGK alterations leading to: - defects in CL biosynthesis → altered OXPHOS - defects in mitochondrial protein import via TIM22 complex
Mt-tRNA defects	MELAS	tRNA^Leu^ (MT-TL1)	Multisystemic syndrome. Mitochondrial myopathy, encephalopathy, lactic acidosis, and cardiomyopathy (HCM or DCM) with stroke-like episodes.	Mitochondrial tRNA defects → decreased expression of OXPHOS components
	MERFF	tRNA^Lys^ (MT-TK)	Multisystemic syndrome. Myoclonus, epilepsy, ataxia, muscle weakness and cardiomyopathy (HCM or DCM).	
Fatty acid oxidation disorders	ß-oxidation defects	*VLCAD, LCHAD, TFP*	Multisystemic disorders. Cardiac manifestations include HCM, arrhythmias, cardiac insufficiency.	Cardiac lipidosis ↓Fatty Acid Oxidation
	Carnitine transporter deficiency	*SCL25A20, CPT2*		
Storage disorders	PRKAG2 cardiac syndrome	*PRKAG2*	HCM, ventricular pre-excitation, supraventricular arrhythmias, atrioventricular block.	↑Activity of AMPK within glycogen metabolism → glycogen accumulation ↑Activity of AMPK unrelated to glycogen → abnormal activation of mTOR pathway
	Pompe disease	*GAA*	Multisystemic disease. Generalized weakness, progressive dysfunction of skeletal and respiratory muscles. Cardiac phenotypes include HCM or DCM.	↓GAA → intra-lysosomal accumulation of glycogen
	Danon disease	*LAMP2*	Multisystemic disorder. HCM and DCM with or without conduction defects, skeletal myopathy, mental retardation.	Glycogen deposit. Accumulation of autophagic vesicles. Mitophagy defects. Altered expression of key mitochondrial genes and impaired mitochondrial respiration.
	Fabry disease	*GLA*	Multisystemic disorder affecting nervous system, kidneys, eyes, skin, and heart. Cardiac phenotypes include: LV hypertrophy, systolic and diastolic dysfunction, myocardial ECM remodeling, valvular abnormalities, vascular and conduction dysfunctions.	↓GLA → Gb3 accumulation in lysosomes → reduced activity of OXPHOS complexes I, IV, V → release of pro-inflammatory cytokines → growth-promoting factors → increased oxidative stress

↑, increased; ↓, decreased; →, triggers

**Table 2:** Examples of signs and symptoms suggestive of specific diagnoses. Image adapted with permission European Society of Cardiology (ESC) Guidelines on Diagnosis and management of hypertrophic cardiomyopathy (HCM) [234].

Symptom/sign	Diagnosis
Learning difficulties, mental retardation	• Mitochondrial diseases
	• Danon disease
Sensorineural deafness	• Mitochondrial diseases
	• Fabry disease
Visual impairment	• Mitochondrial diseases (retinal disease, optic nerve atrophy)
	• Danon disease (retinitis pigmentosa)
	• Fabry disease (cataracts, corneal opacities)
Gait disturbance	• Friedreich’s ataxia
Paraesthesia/sensory abnormalities/neuropathic pain	• Fabry disease
Muscle weakness	• Mitochondrial diseases
	• Glycogen storage disorders
	• Friedreich’s ataxia
Palpebral ptosis	• Mitochondrial diseases
Angiokeratomata, hypohidrosis	• Fabry disease
